# Ulnar-Basilic Arteriovenous Fistula for Hemodialysis Access: Utility as the “Second Procedure” after Radio Cephalic Fistula

**DOI:** 10.3400/avd.oa.20-00124

**Published:** 2021-06-25

**Authors:** Shobhit Sharma, Sudipta Bera, Vikas Deep Goyal, Vivek Gupta, Navneeta Bisht

**Affiliations:** 1 Department of Surgery, Shri Ram Murti Samarak Institute of Medical Sciences, Bareilly (UP)-243202, India; 2 Department of Plastic and Reconstructive Surgery, Institute of Medical Sciences, Banaras Hindu University, Varanasi (UP), India; 3 Department of Plastic and Reconstructive Surgery, Sir Gangaram Hospital, New Delhi, India; 4 Department of Anesthesia, Shri Ram Murti Samarak Institute of Medical Sciences, Bareilly (UP), India

**Keywords:** ulnar-basilic, radio cephalic, arteriovenous fistula, hemodialysis, vascular access

## Abstract

**Objectives**: As per standard guidelines, the recommended order of arteriovenous fistula (AVF) creation for hemodialysis (HD) access is radiocephalic (RC), followed by proximal elbow fistulas and arteriovenous graft. Although ulnar-basilic (UB) fistula has been an alternative to RC-AVF, still this procedure searches clear recommendations. We present here our experience on UB-AVF as the preferred “second procedure” instead of proximal fistula after the RC-AVF.

**Methods**: Forty-two UB-AVF were created in nonfeasible and failed RC-AVF cases between 2016 and 2018. They were reviewed retrospectively and outcomes were compared with 480 RC-AVF constructed within the same period.

**Results**: The primary patency at 18 months was 73.8%, 69.6% and mean maturation time was 33.7±6.6 days, 32.1±4.7 days for UB-AVF and RC-AVF respectively (p>0.05).

**Conclusion**: Our altered order of preference enabled us to create all the first-time fistula in the distal forearm, providing all the advantages of distal fistula like RC-AVF and avoiding proximal fistula, improved patient convenience and short-term benefit. In an inference that may be used for references and needs support from a larger sample and longer duration study from other centers, UB-AVF may be considered as the second option after RC-AVF depending on the clinical scenario.

## Introduction

As per the Kidney Disease Outcome Quality Initiative of the National Kidney Foundation (NKF-KDOQI), and most of the other guidelines, the recommended order of arteriovenous fistula (AVF) creation for hemodialysis (HD) access are: radiocephalic (RC); brachiocephalic (BC); and transposed brachial-basilic vein fistula followed by arteriovenous graft of biological or synthetic material.^1–3)^ Native distal forearm RC-AVF is the gold standard for technical simplicity, and lower complication rate. But sometimes distal forearm fistula creation is marred by small-caliber vessels and thrombosed cephalic veins from prior cannulation lead to choice for a proximal fistula. Although proximal fistula provides better flow and faster maturation, it is known for technical complexity and higher complications such as hand edema, steal phenomenon, high output cardiac failure, etc.^2,4,5)^ Ulnar-basilic(UB) fistula has been described as an alternative procedure in recent studies, but still this procedure searches clear recommendations.

We present here our experience on UB-AVF as the preferred “second procedure” instead of proximal fistula after the RC-AVF.

### Aims and objectives

We aim to assess the utility of UB-AVF for distal forearm fistula creation according to our preferred order of fistula creation.

## Materials and Methods

This study includes patients referred from the nephrology department between 2016 and 2018 for AVF creation. The patients in whom UB-AVF were created were reviewed retrospectively, and the outcome is compared with the RC-AVF created within this period by us. Patient-related variables are collected from the hospital database and subjected to statistical analysis.

### Selection criteria

Patients were assessed clinically and with color Doppler study. Patients were selected for UB-AVF in the following situations.

1. Nonfeasibility of RC-AVF as radial artery or cephalic vein diameter <1.5 mm in the mid and distal forearm and a dominant basilic venous system.
2. Failed RC-AVF in both forearms.
3. Failed RC-AVF in the nondominant forearm in young and working individuals. In these cases, the dominant forearm is spared for functional use.
4. The cephalic vein is thrombosed from prior cannulation.

Prerequisite for a UB-AVF was ulnar artery and basilic vein diameter >1.5 mm on Doppler study with a clinically distensible basilic vein. We have followed the overall sequence for surgical fistula creation suggested by NKF-KDOQI guidelines. However, while also chosing to spare the dominant forearm from first-time fistula creation in young and working individuals and observing quite a good number of patients requiring fistula in this group, we preferred, based on our previous experiences on UB-AVF and evidences in literature,^6–8)^ to choose a distal site over proximal for first-time fistula creation. We have followed the same sequence throughout the study period. However, patients on prolonged catheter dialysis are preferred for proximal fistula instead of UB fistula.

### Preoperative preparation

The patients were examined clinically at the office with a tourniquet applied above the elbow. Venous distensibility and course were noted. A color Doppler assessment of the forearm and arm of both limbs was done. An Allen test was performed to assess the dual vascularity of hand. In previous failed RC-AVF cases, Doppler assisted Allen test supplemented clinical assessment and the extension of thrombus noted. Patients were selected for fistula creation according to clinical and color Doppler findings. Patients fulfilling the selection criteria for UB fistula were taken for surgery. All surgeries were performed by the same team of surgeons (SS and SB) on the same day or next day as an outpatient procedure.

All the first-time fistula was created in a single consultation. In previously failed cases fistula was created after two weeks of previous surgery. In dialysis-dependent cases, heparin-dialysis was resumed after six hours of fistula creation.

### Operative technique

**Figure 1:**
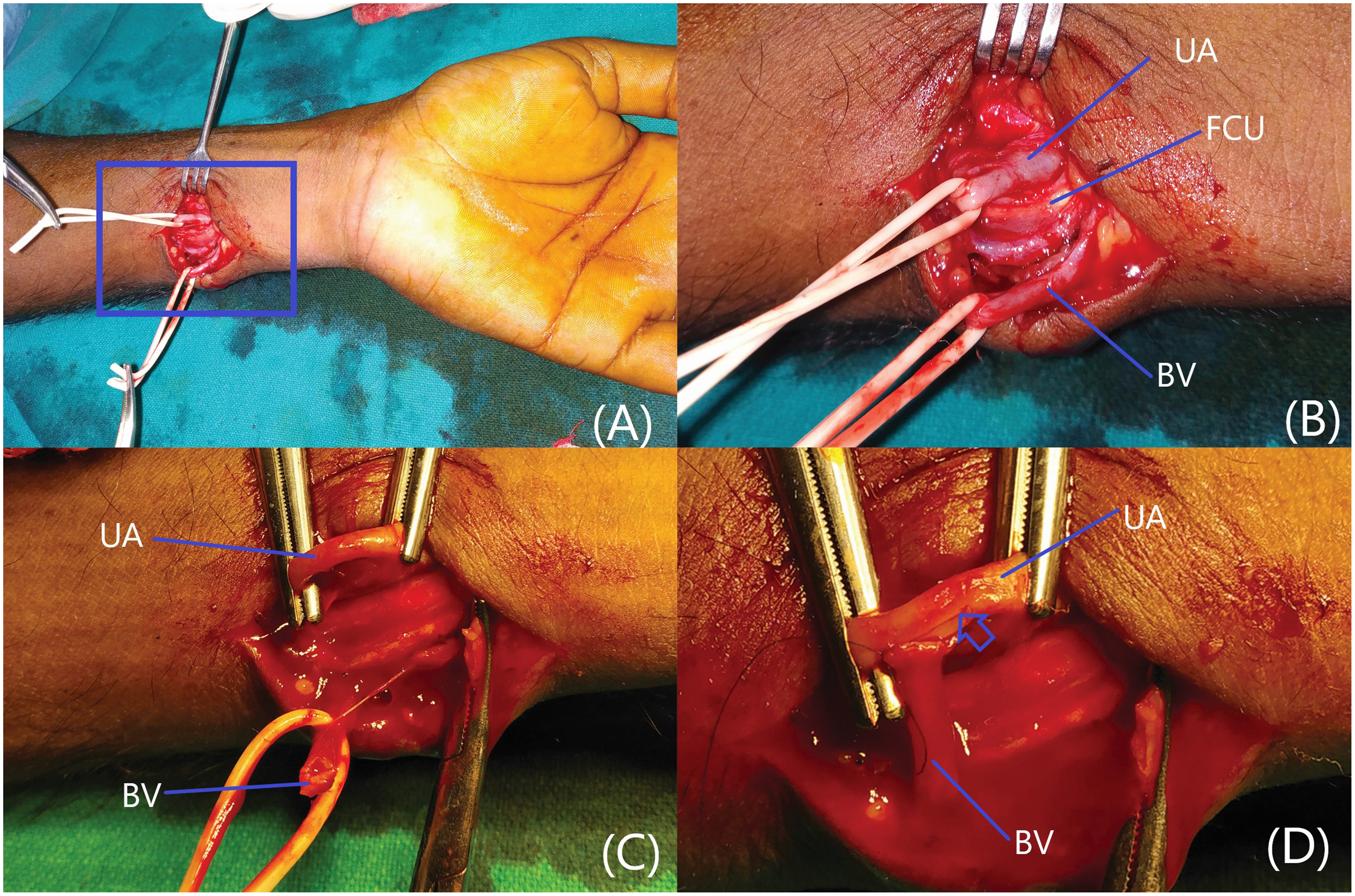
Fig. 1 Exploration of Vessels for UB-AVF creation. (**A**) Exploration of vessels at the distal forearm. (**B**) Zoomed view of the operative field. (**C**) Venous rent prepared for anastomosis. (**D**) Arteriotomy and matching of vessel caliber. The arteriotomy site is indicated with the blue arrow. UB-AVF: ulnar-basilic arteriovenous fistula; UA: ulnar artery; FCU: tendon, and distal muscle belly of Flexor Carpi Ulnaris; BV: basilic vein

**Figure 2:**
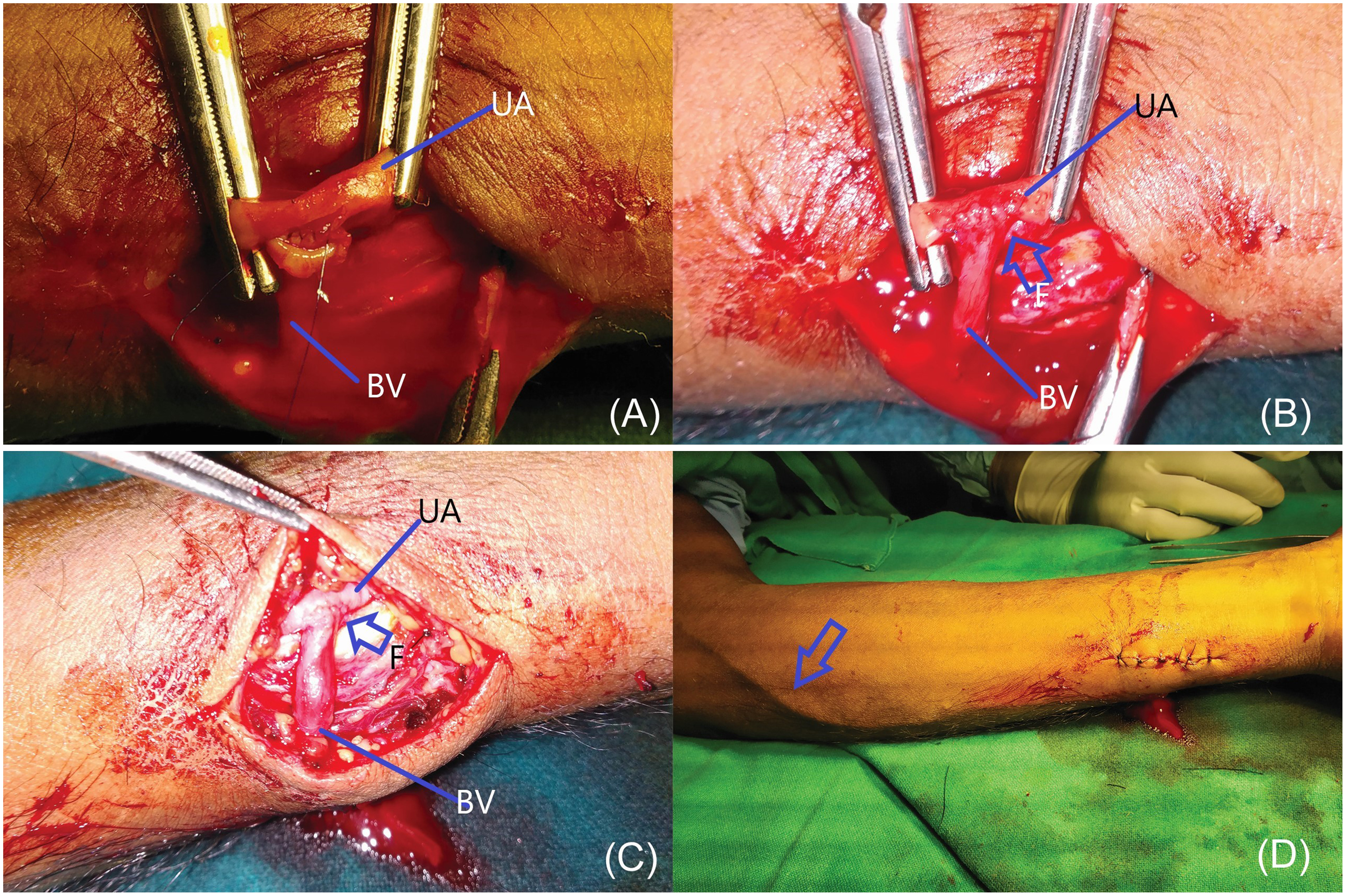
Fig. 2 UB-AVF creation. (**A**) Arteriovenous anastomosis starting from the posterior wall. (**B**) Anastomosis before releasing the clamps. (**C**) Establishment of fistula. (**D**) Incision closure. The immediate venous dilatation at anastomotic site and forearm can be appreciated from (**C**) and (**D**).

UB-AVF: ulnar-basilic arteriovenous fistula; UA: ulnar artery; BV: basilic vein; F: fistula

All the surgeries were done by two surgeons (SS and or SB under observation of SS) and the preoperative assessment was done by either of them. The surgeries were performed under local anesthesia with field infiltration of 2% plain Xylocaine and loupe magnification. A longitudinal skin incision was created on the ulnar aspect about 5–6 cm proximal to the wrist centering the proposed fistula site as per clinical and Doppler findings. The basilic vein was exposed and assessed for suitability for fistula creation. The vein was mobilized and the venous bifurcation was used as a “branch patch” whenever available. The deep fascia of the forearm was incised, the Flexor Carpi Ulnaris (FCU) tendon was identified and retracted toward the ulnar side. The ulnar artery was identified deep and radially to this tendon and separated from its venae commitantes and ulnar nerve (**Figs. 1A** and **1B**). Special care was taken to avoid ulnar nerve injury, which lies in the groove between Artery and FCU tendon. Ulnar perforator branches were ligated. The ulnar artery was elevated in between a pair of hemostatic clamps. The basilic vein was divided, venous rent was prepared and mobilized to the proposed arteriotomy site. An arteriotomy was made on the lateral wall of the ulnar artery in between hemostatic clamps. The arteriotomy length is adjusted to match venous rent and was kept approximately 8–12 mm, to avoid too wide fistula (**Figs. 1C** and **1D**). An end to side anastomosis was made with continuous 7–0 Prolene suture (**Figs. 2A** and **2B**). On completion of the anastomosis, hemostatic clamps were released and fistula was established (**Fig. 2C**). Immediate post-operative venous dilatation and palpable thrill at the anastomotic site were checked (**Fig. 2D**). Hemostasis was checked, any additional large venous branch was ligated and the wound was closed in a single layer with nonabsorbable suture. The operated hand was kept elevated, warm and bruit and thrill reassessed half an hour later and finally before discharge. The patients were discharged with oral antibiotics and paracetamol analgesia for five days.

### Postoperative care

The patients were reviewed in the office on 2nd postoperative day. The bruit and thrill were reassessed clinically and with a handheld Doppler. No re-exploration was done unless there is an expanding lesion at the operative site. The patients were followed up after a week and sutures were removed.

### Hemodialysis

Patients were followed up at the nephrology department subsequently and in dialysis dependent patients catheter dialysis was resumed six hours after operation till fistula matures. Pre dialysis fistulas were assessed clinically and with the color Doppler study. Palpable thrill in a >5 cm dilated segment of vein was assessed with a Doppler study for flow rate. Patients were cannulated in flexed elbow position and when a flow rate of 250 ml/min was attained, dialysis was initiated through it. Fistula maturation time was noted.

### Follow up

Patients were followed up as and when required onwards and fistula patency was checked every time. Patients were considered for alternative procedures if thrill did not progress or maturation did not attain after a maximum 6–8 weeks follow up, with patients being subjected to reoperation if fistula was found to be blocked as evident by color Doppler study. No secondary intervention was done due to the lack of a cost-effective radiologic intervention facility at our center.

### Statistical analysis

All data were tabulated and were analyzed with Graph Pad Prism version 5 Software. Continuous data are expressed as with mean±standard deviation (SD), and comparisons were made using the unpaired t-test. Nominal or ordinal variables were expressed as frequency and percentage (%), and compared using the Chi-square (χ^2^) test. P-value <0.05 was considered as significant. Fistula primary patency rates were estimated by the Kaplan–Meier method and the curves are compared with the Log-rank test.

### Ethics

This study complied with the standards of the Declaration of Helsinki and current ethical guidelines. It was approved by the institutional ethics board at the Sri Ram Murti Smarak Hospital and Institute of Medical Sciences, India (E-1847). Written informed consent was obtained from all the patients before each surgical procedure and for the utilization of data for publication.

## Results

Total of 42 UB-AVF was created within the study period. The mean age of the patients was 55.8±10.5 years. Among the cases, 12 (28.6%) were female, and 30 (71.4%) were male. Among the 480 RC-AVF cases, 304 (63.3%) were male and 176 (36.7%) were female and the mean age of the patients was 55.5±9.8 years. The groups were comparable according to age (p=0.9) and sex (p=0.3) distribution without any statistically significant difference. All the UB fistula was created in the distal forearm within 12 cm from the proximal wrist crease. Thirty-seven (88.1%) fistula was constructed on the nondominant forearm. Among the fistula 31 (73.80%) were created as the first-time fistula in nonfeasible RC-AVF and 11 (26.19%) in previously failed cases. Thirty-six patients were HD dependent, on catheter dialysis and six were pre dialysis.

Mean ulnar artery and basilic vein diameter was 2.4±0.3 mm, and 2.348±0.4 mm, respectively. The mean operative time was 53.8±8.8 min. Fistula maturation time to dialysis was 33.7±6.6 days.

**Figure 3:**
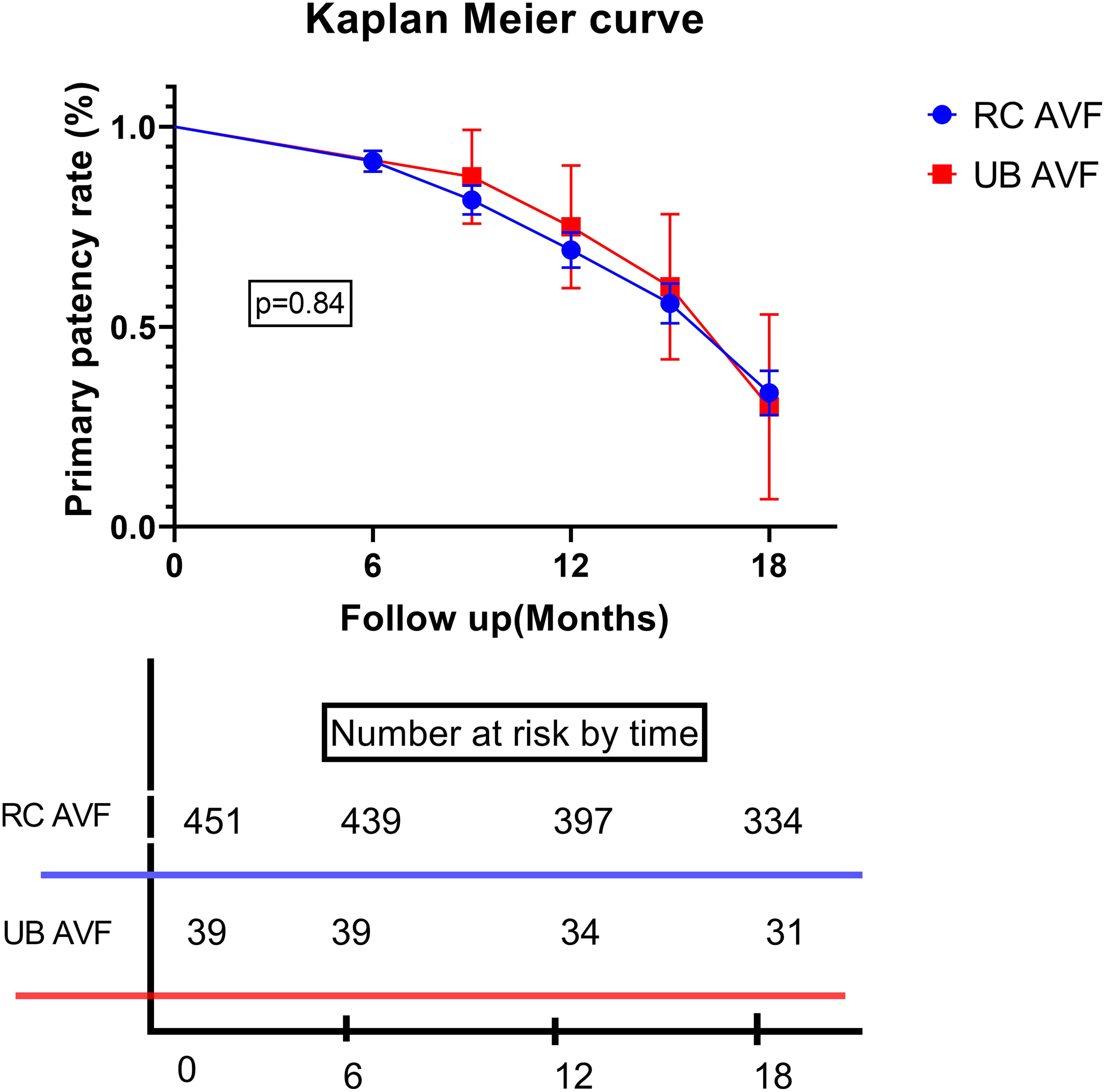
Fig. 3 Survival curves (Kaplan–Meier) for primary patency in patients with ulnar-basilic arteriovenous fistula (UB-AVF) and radiocephalic arteriovenous fistula (RC-AVF).

The outcome is compared with 480 RC-AVF created as per the preference protocol within the same period (**Table 1**). Kaplan–Meier curves for the primary patency rate of RC-AVF and UB-AVF are shown in **Fig. 3**. There was no significant difference of primary patency rate between the groups (p=0.84).

**Table 1:** Table 1 Comparative outcome of RC-AVF and UB-AVF

Parameters	RC-AVF(n=480)	UB-AVF(n=42)	p/χ^2^ value
Thrombosis	21 (4.4%)	0	p=0.2, χ^2^=1.8 (NS)
Failure to mature	8 (1.7%)	3 (7.1%)	p=0.2, χ^2^=5.2 (NS)
Primary patency to HD	451 (93.9%)	39 (92.9%)	p=0.78, χ^2^=0.081 (NS)
Maturation time (days)	32.1 (± 4.7)[n=451]	33.7±6.7 [n=39]	p=0.2 (NS)
Primary patency/failure rate at 18 Months	334 (69.6%)/60 (12.5%)	31 (73.8%)/4 (9.5%)	p=0.6, χ^2^=0.4 (NS)

RC-AVF: radiocephalic arteriovenous fistula; UB-AVF: ulnar-basilic arteriovenous fistula; HD: hemodialysis; NS: Not significant

### Complications

While hand edema, steal phenomenon and ischemic manifestations, ulnar nerve injury and other complications related to surgery were not seen in any of the cases, extravasation was noted in one case, which was managed conservatively and fistula remained patent after swelling subsided.

## Discussion

As per standard guidelines, RC-AVF is the gold standard vascular access for HD. Distal forearm native fistula is always a preferred approach due to technical simplicity and preservation of proximal venous capital. UB-AVF was first described by Hanson et al.^9)^ in 1967, almost at the same time as Bresica et al.^6)^ reported RC-AVF. But UB-AVF went out of favor after the initial high failure rate and prolonged maturation time.^10)^

Where UB-AVF has been considered as an alternative to RC-AVF, and a better utility as first-time procedure than proximal fistula has been established in the present literature,^5,11–15)^ several case series and meta-analysis have been published in subsequent years and remarkable improvement of success rate has been reported in these studies.^6,16,17)^ However, still these findings need further shreds of evidence to include it in the recommended guidelines.

The ulnar artery has almost similar course and caliber in the distal forearm as the radial artery. Moreover, the basilic vein is usually escapes cannulation and thrombosis due to its unfavorable location. Thus UB-AVF remains a viable alternative to RC-AVF with unfavorable vessels. The basilic vein is reported to have a thinner wall than the cephalic vein, where, in contrast to RC-AVF, there are technical difficulties related to mobilizing the artery from the deeper surface to FCU tendon and proximity to the ulnar nerve, which is the main motor nerve for hand function.

The major drawbacks of this procedure are technical difficulty in handling smaller caliber vessels, thin basilic vein, and difficult anastomosis, kinking of fistula while arising from deep to FCU tendon, longer maturation time, and difficult cannulation for HD. However, the procedure has been described in these literatures to be safe, and complications are minimal.^8,10,15,16)^ Thus the major merit of the procedure lies in having all advantages of distal fistula creation and a better complication profile than proximal fistulas. Moreover, as the proximal venous capital is preserved, the impact of failure is minimal.

**Figure 4:**
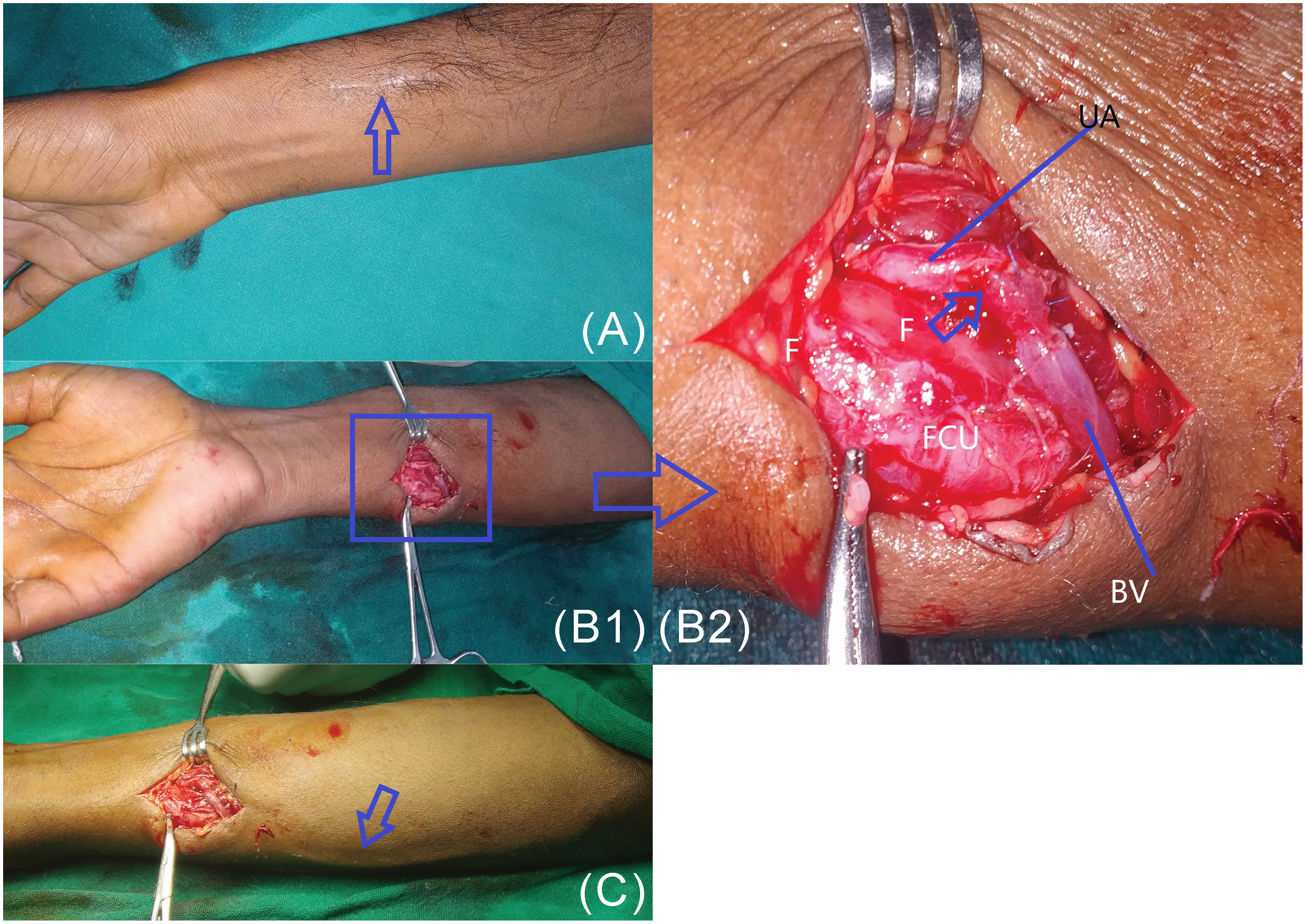
Fig. 4 UB-AVF in previously failed RC-AVF. (**A**) Site of the previous fistula. (**B1** & **B2**) Fistula at the distal forearm, deep to FCU muscle. (**C**) Immediate notable venous dilatation. UB-AVF: ulnar-basilic arteriovenous fistula; RC-AVF: radiocephalic arteriovenous fistula; UA: ulnar artery; FCU: muscle belly of Flexor Carpi Ulnaris; BV: basilic vein; F: fistula

Our study reports UB-AVF creation in patients with unfavorable cephalic vein at wrist and forearm and favorable ulnar artery and brachial vein configurations. Based on recent evidences, we explored the option of UB-AVF for time fistula creation in nonfeasible RC-AVF, and preferred fistula creation on the nondominant forearm. We also preferred the procedure over proximal fistula in failed RC-AVF. UB-AVF was created on the same limb after clinical and Doppler assessment in these cases. We have used a similar method as our preferred way of anastomosis for both UB-AVF and RC-AVF.^18,19)^ An end to side anastomosis with ligation of distal basilic vein was done. The “Brach patch”^20)^ was used whenever venous bifurcation was available. Fistula had an oblique course over the convexity of FCU muscle at its origin and kinking during the change of course was not seen in any case (**Fig. 4**). We have observed the technical simplicity comparable to RC-AVF creation. Mean operative time was 53.8±8.8 min, which was similar to the operative time of an average RC-AVF creation. Mean venous and arterial diameter was (2.3±0.4) mm and (2.4±0.3), respectively, and we have not faced any difficulty in anastomoses under loupe magnification.

The outcome of UB fistula created with similar techniques for RC-AVF, and preference protocol is compared with the fistula outcome of our RC-AVF cases. Our results show an almost equal success rate to the RC-AFV with primary patency to maturation rate of 93.9% and 92.9% for RC-AVF and UB-AVF, respectively. The unassisted primary patency at six months, 12 months, and 18 months were 39 (92.9%), 36 (85.7%), and 31 (73.8%), respectively. The outcome was similar to RC-AVF, and there were no statistically significant differences in the survival rate. This patency rate was similar to the reported improved patency rate in recent studies also^6,16,17)^ (**Table 2**).

**Table 2:** Table 2 Outcome of ulnar-basilic arteriovenous fistula in literature

Author	Primary patency at 1 year	Maturation time	Number of fistula	Functional maturation
Fonseka 2002		6–10 weeks	10	100%
Salgado 2004	70.9%	≥8 weeks	61	91.9%
Weyde 2008	70.4%		13	
Cavatorta 2008	78%		9	
Hiroshi 2010	45.1%		44	
Bourquelot 2011	42%	80 days	70	60%
Liu 2013	43%	100 (32–471) days	52	36%
Shintaku 2014	25%	20±7 days	29	
Al Sakarchi 2016	53.0%		Meta-analysis of 8 studies	
Schwein 2016		66 days	33	60%
Zhen 2017	77.2%		44	
Present study	73.8%	33.7±6.6 days	42	92.9%

The mean fistula maturation time was 33.7±6.6 days, which is similar to our RC-AVF cases (32.2±4.7 days, p=0.166, not significant). We have noted better maturation time than the earlier reports showing longer maturation time as a major drawback of UB fistula.^10,16)^ The maturation time in our study fulfills the recommended limit of six weeks for RC-AVF maturation.^3)^ This better maturation profile may be attributed to the anastomosis technique, vessel diameter, and selection of cases.^21,22)^ It has been reported that an increased flow rate and diameter has been observed in the ulnar artery when the radial artery is not functioning.^6,8,23)^ In our series we have obtained a suitable or better size ulnar and or, the basilic vessel in previously failed cases also. In 11 patients with previously failed RC fistula, UB-AVF was created in the same forearm. While in five cases complete occlusion was noted but color Doppler assisted Allen test indicated maintained dual vascularity by increased flow through the interosseous artery, although the RC-AVF failed due to thrombosis, the thrombosis did not extend to the radial artery in six cases and an Allen test indicated preserved good dual vascularity of hand. All the fistula remained patent at one-year follow up. Remarkably, hand edema and ischemic complications have not been noted in any of these cases. We have not observed any major complications related to surgery other than extravasation in one case (1/42).

The major concern was difficulty in cannulation for HD. But this was improved with cannulation and care by efficient dialysis personnel. This did not have any major drawback on surgical outcomes.

Thus our altered order of preference added a short term benefit to patients and treatment in our institute with an overall reduction of cost and inconvenience. Our preferred order enabled us to achieve distal forearm fistula creation in all cases with acceptable primary patency, fistula maturation rate, and without any major complications, and all the cases were done comfortably as an office procedure. The creation of proximal fistula and related complications was avoided effectively, even in previously failed fistula also.

## Conclusion

Our study reports an almost similar outcome as of our RC-AVF cases. While providing all the benefits of distal fistula like RC-AVF and avoiding proximal fistula improved patient convenience and short term patient benefit in selected situations, our altered order of preference enabled us to create the first-time fistula in the distal forearm in all cases and also as the second procedure in previously failed cases. Thus UB-AVF may be considered as the second option in the favored order for fistula creation depending on the clinical scenario. This inference may be used for references and needs support from a larger sample and longer duration study from other centers to include this in the recommended protocol.

### Limitations

The success rate of this study is reflected by the number of usable fistula for dialysis, maturation time, and lower complication rate, where the study is limited by a short follow-up period due to major loss in follow up once dialysis has been started through the successful fistula. The study also did not include the benefit of secondary interventions due to the lack of cost-effective radiologic intervention facilities at our center.
